# Novel surgical approach to nasal dorsum midline dermoid sinus cyst

**DOI:** 10.1080/23320885.2023.2256398

**Published:** 2023-09-11

**Authors:** Aynur Aliyeva

**Affiliations:** a Otolaryngology Specialist, Azerbaijan Medical University, Baku, Azerbaijan; b Department of Neuroscience, Yeditepe University, Istanbul, Turkey

**Keywords:** Nasal dermoid sinus cyst, septal cartilage, novel technique

## Abstract

The correct reconstructive technique following dermoid cyst removal is critical for treatment and aesthetics. This article will discuss the management of nasal dermoid sinus cysts. A mixture of “Turkish-delight” crushed cartilage with Fibrin-based tissue adhesives and non-crushed septal cartilage was used for the primary reconstruction of the nasal dorsum.

## HIGHLIGHTS

1. **Nasal dermoid cysts**: Nasal dermoid sinus cysts are rare midline abnormalities that can occur on the nasal dorsum, typically between the glabella and the columella. Surgical intervention is usually necessary to prevent complications and ensure complete removal of the cyst.
2. **Surgical approaches:** Various surgical approaches can be used for the treatment of nasal dermoid cysts, including excision with primary closure, midline vertical incision, transverse incision, lateral rhinotomy, inverted-U incision, and external rhinoplasty. The choice of approach depends on factors such as the size, location, and extent of the cyst, as well as the patient’s condition and the surgeon’s preference.
3. **Importance of complete excision**: Complete excision of nasal dermoid cysts is crucial to prevent complications such as recurrence, nasal deformity, infection, meningitis, and intracranial abscesses. Proper surgical management aims to remove the cyst and associated sinus tracts while restoring normal nasal anatomy and function.
4. **Surgical technique**: In a case study involving a 73-year-old male patient with a nasal dermoid cyst, an inverted T-shaped incision was made on the nasal dorsum. The entire cystic mass was carefully removed along the midline, and any eroded nasal dorsum bones were drilled to create a single common cavity. The defect on the nasal dorsum was repaired using a combination of crushed septal cartilage mixed with Fibrin-based tissue adhesives and small cartilaginous palisade grafts for aesthetic reconstruction.
5. **Successful outcome**: The patient underwent successful surgery with no recurrence observed during the one-year follow-up. The wound site healed completely, and the patient maintained a pleasing profile. This case highlights the effectiveness of the surgical technique in managing nasal dermoid cysts while minimizing defects and achieving aesthetic and functional outcomes.

## 1. Introduction

Teratomas are a type of tumor that arises from the three embryonic germ layers: the mesoderm, endoderm, and ectoderm [1]. These tumors can occur in various tissues throughout the body [2,3]. Teratoid tumors and dermoid cysts located on the face are more frequently observed in the midline. Unlike craniofacial dermoids, they may take the form of cysts, sinuses, or fistulas, but rarely, they can extend intracranially [4,5]. These tumors are typically symptomatic during childhood, and although they rarely become malignant, surgical intervention is the primary treatment approach). NDSCs (Nasal Dermoid Sinus Cysts) can present as a cystic mass or sinus opening on the midline nasal dorsum, typically between the glabella and the columella at birth or during early childhood [3,5].

Complete excision of NDSCs is crucial in order to prevent complications such as recurrence, nasal deformity, infection, meningitis, and the formation of intracranial abscesses. Several surgical approaches are utilized for the treatment of NDSCs. Proper surgical management aims to achieve complete removal of the cyst and its associated sinus tracts and restore normal nasal anatomy and function [6–10].

Introducing this surgical technique into the existing literature aims to raise awareness about the potential occurrence of nasal dermoid cysts on the midline nasal dorsum, even in advanced age. Furthermore, it underscores that effective management of such cases with minimal defects is achievable through meticulous surgical strategizing. The primary objective is to present a successful case of managing a dermoid cyst in the middle nasal dermal sinus.

The treatment strategy employed a combination of two materials: “Turkish-delight” crushed cartilage and Fibrin-based tissue adhesives, which were utilized as a matrix to fill the excised area. In addition, non-crushed septal cartilage was employed for the primary reconstruction of the nasal dorsum. This innovative approach is highlighted to showcase its positive outcome and to emphasize its potential for addressing similar cases in the future.

## 2. Surgical technique

Following radiological imaging, a 73-year-old male patient with a history of progressive swelling on the nasal dorsum for the past year was diagnosed with a nasal dermoid cyst. Nasal endoscopy findings were normal, and subsequent brain and paranasal sinus tomography and clinical examinations did not reveal any signs of intracranial extension. Consent to publish this case report has been obtained from the patient in writing.

**Figure 1.**
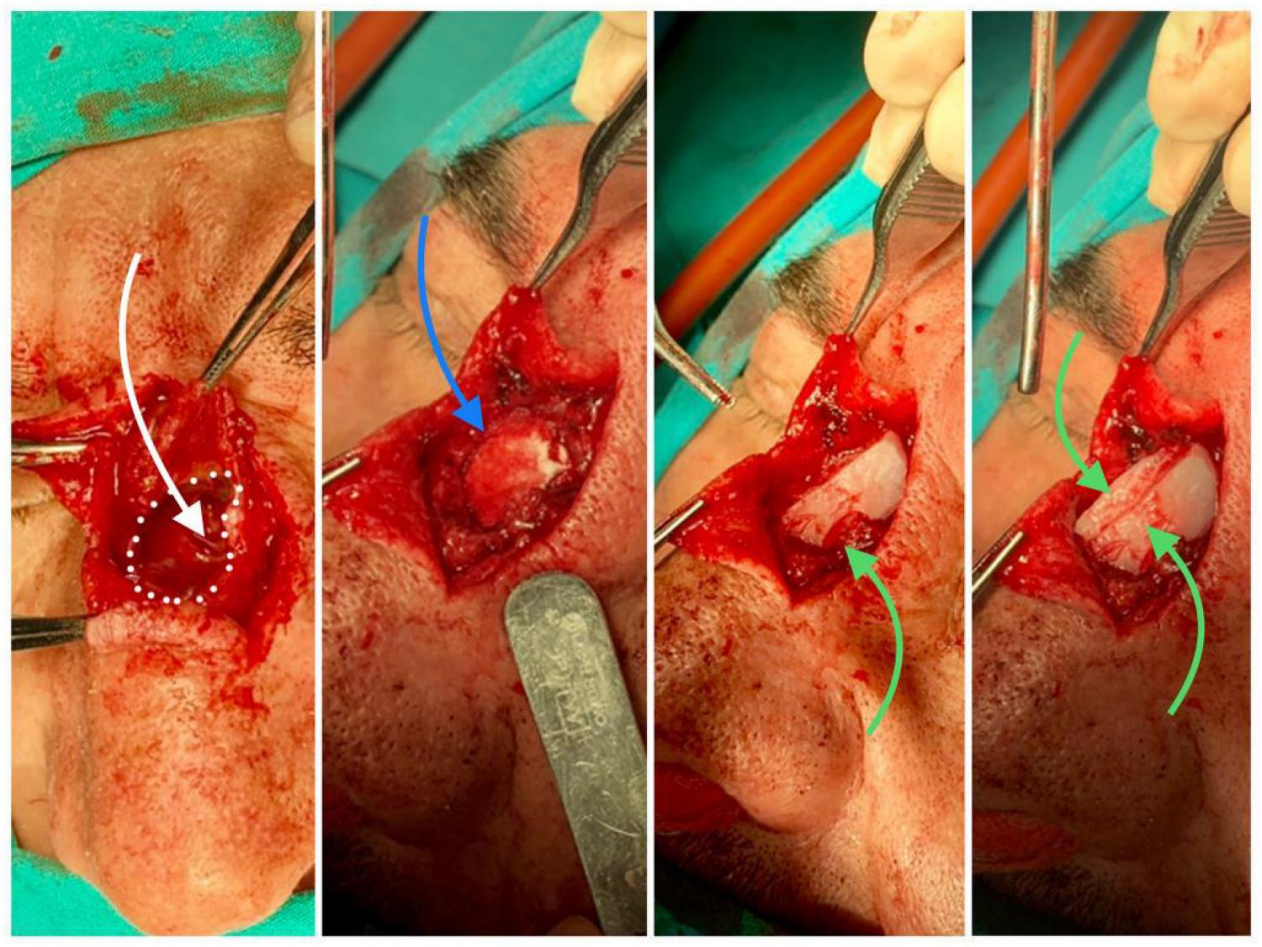
Surgical technique. White dots and white arrow: Erosion was observed in the lateral left nasal bone, middle ethmoid cells, and upper septum. Blue arrow: The harvested septal cartilage sections resembling “Turkish delight” were mixed with Fibrin-based tissue adhesives, forming a mixture that was applied to fill the defect on the nasal dorsum. Green arrow: Two small septal cartilage palisade grafts were placed over the nasal septal defect to cover the nasal dorsum defect and achieve a more aesthetically pleasing profile.

The patient underwent surgery under general anesthesia. The initial step involved deciding on the approach and the type of incision. An inverted T-shaped incision was made on the nasal dorsum, followed by skin and subcutaneous tissue dissection to access the cystic mass. Careful removal of the entire cystic mass was performed along the midline of the nasal dorsum. Erosion was observed in the lateral left nasal bone, middle ethmoid cells, and upper part of the septum. The eroded portion of the nasal dorsum bones was drilled to create a single common cavity (Figure 1. White dots and white arrow). The patient’s septal cartilage was excised to repair the tissue defect on the nasal dorsum. The nasal septum was then reshaped into two parts: one part was harvested as small sections resembling “Turkish delight,” while the second part was divided into two small cartilaginous palisade grafts measuring approximately 1 cm × 1.5 cm each. The harvested septal cartilage sections resembling “Turkish delight” were mixed with Fibrin-based tissue adhesives, forming a mixture that was applied to fill the defect on the nasal dorsum (Figure 1. Blue arrow).

**Figure 2.**
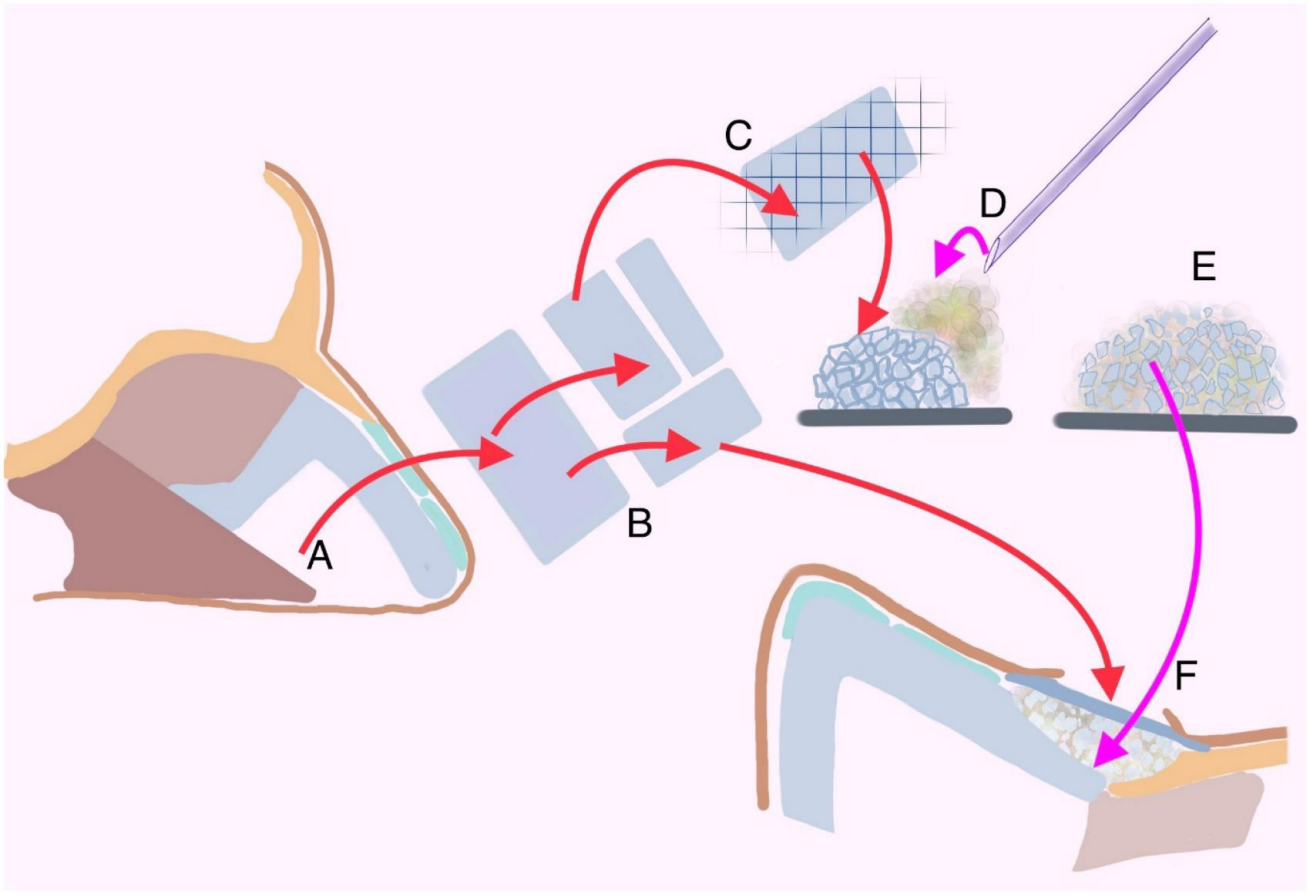
Illustration about the concept of the surgery defect repairing with ‘’Turkish delight’’ and fibrin glue mixture, covering with palisade cartilage grafts. (A) Resection of a singular quadrilateral cartilage piece measuring 1.5 × 2 cm from the septal cartilage. (B) Subdivision of the septal cartilage graft into petite palisade cartilage grafts with sleek surfaces using a scalpel. (C) Partitioning of the cartilage graft into minuscule particles measuring 0.1 × 0.2 cm, termed as 'Turkish delight.' (D) Application of tissue fibrin glue onto the compacted mass of 'Turkish delight’ cartilage. (E) Homogenization of the 'Turkish delight’ cartilages by blending them with fibrin tissue glue to attain a uniform mixture. (F) Placement of the resultant mixture into the post-excision surgical defect (nasal dorsum), followed by positioning of cartilage palisades onto the mixture to enhance the natural contour of the nasal dorsum.

**Figure 3.**
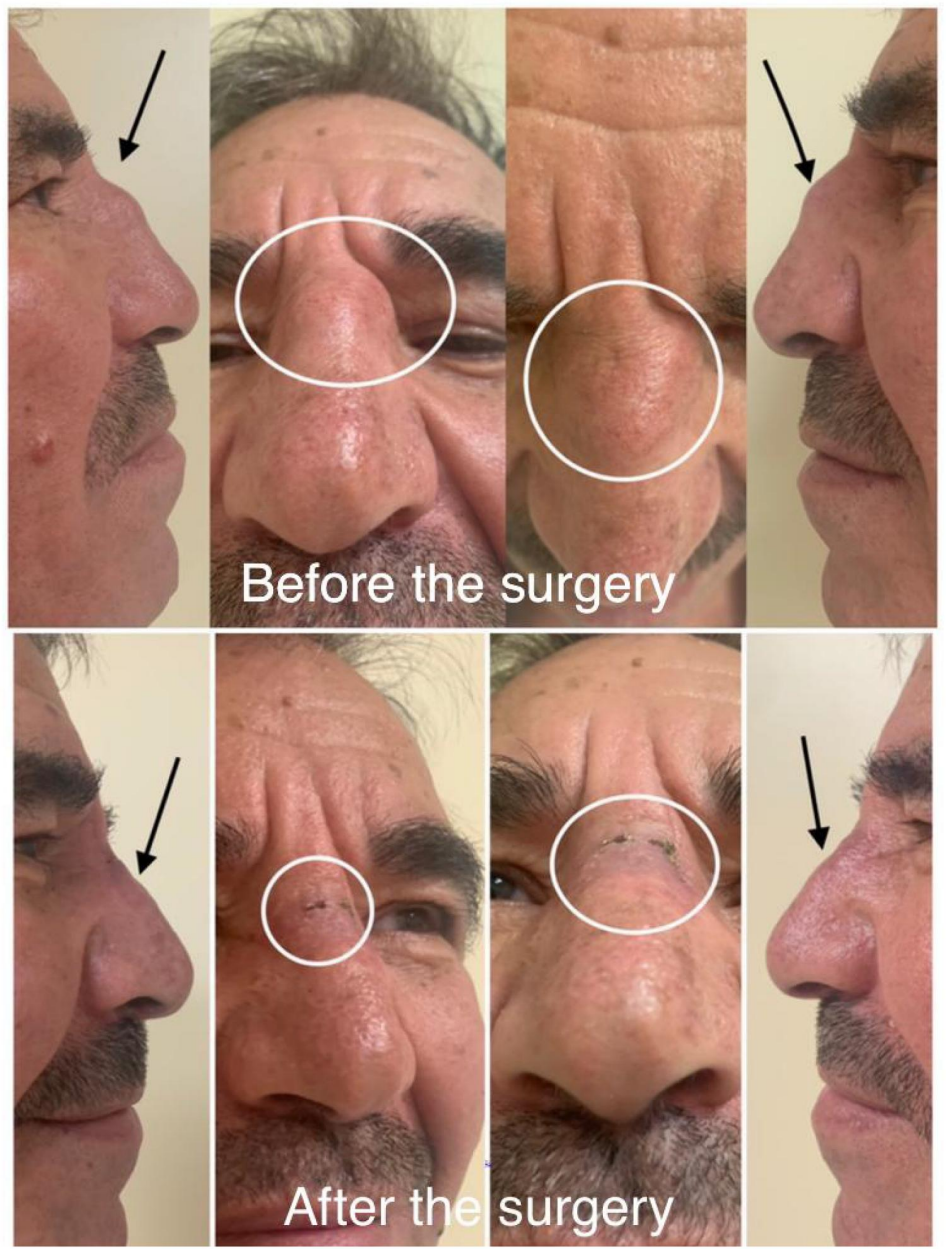
Photos from different views.

Additionally, the two small septal cartilage palisade grafts were placed over the nasal septal defect to cover the nasal dorsum defect and achieve a more aesthetically pleasing profile (Figure 1. Green arrow). The dorsum defect was nearly completely repaired, and the T-shaped incision was closed without any noticeable defects. All concepts of the “Turkish delight and fibrin glue mixture with the palisade cartilage grafts are shown in the illustration from Figure 2. In the patient’s one-year follow-up, no recurrence was observed. The wound site healed completely, and the patient continued to use glasses (Figure 3).

## 3. Discussion

Cystic masses are frequently found in the head and neck area due to various reasons like development, infection, tumors, and trauma. However, Epidermoid and Dermoid Cystes are rarely encountered, making up just 3.7% to 11% of congenital masses and less than 10% of head and neck tumors. Typically associated with the orbital region in about half of the cases, our patients showed a different pattern, with nearly equal distribution among the orbital, auricular, and neck regions, and around 20% of cases occurring in other locations [4]. Differentiating various types of cutaneous cysts in the head and neck region is very important for clinicians. This differentiation is crucial due to the diverse nature of these cysts, arising from distinct underlying causes and exhibiting varied clinical presentations. There are different types of cystes based on their pathogenetic mechanisms, such as developmental, infective, neoplastic, and traumatic factors. Each of these types has unique characteristics that aid in differentiation. For instance, developmental cysts like epidermoid and dermoid cysts originate from remnants of embryonic tissue and contain skin components like hair, sebum, and sweat glands. These cysts are often located along lines of embryonic fusion.

Infective cysts, on the other hand, could arise due to infections or inflammatory processes. Clinical features such as redness, warmth, and tenderness may be indicative of an infective origin. Neoplastic cysts, which could be benign or malignant, require careful evaluation for features suggesting malignancy, such as rapid growth, irregular borders, and associated pain. Traumatic cysts might form in response to physical trauma and could be filled with serous or blood-tinged fluid. The location of the cysts can also aid in differentiation. For example, the orbital region is often linked to epidermoid and dermoid cysts, while other regions such as the auricular and neck regions might also be affected, as noted in the study. Histopathological examination is a crucial tool for differentiation. The cyst’s content, cellular composition, and surrounding tissue characteristics can provide important diagnostic information. Molecular markers and imaging techniques could further contribute to accurate differentiation. The study by Al-Khateeb et al. emphasizes the importance of considering the diverse etiologies and clinical presentations of cutaneous cysts in the head and neck region. By carefully analyzing clinical features, location, histopathological findings, and possibly molecular markers, healthcare professionals can effectively differentiate between various types of cutaneous cysts and provide appropriate management strategies for each case [5].

The definitive treatment of nasal dermoid cysts is surgery, and if there is an intracranial extension, excision with craniotomy should be performed. Recurrence was reported as 1–4% after treatment [1,3,6–10]. The external and endoscopic approaches are two commonly used surgical techniques [7–10]. The external approach, also known as an open approach or external rhinoplasty, involves making an incision on the skin surface of the nose. This technique is usually employed when the cyst is large, deeply located, or extends into the nasal bones or frontal sinus [8,9]. The endoscopic approach is a minimally invasive technique that utilizes an endoscope, a thin, flexible tube with a light and camera, to visualize and access the nasal cavity. This approach is typically preferred for smaller, superficial cysts [7,10]. Both techniques have advantages and considerations, and the choice of approach depends on various factors such as the size, location, and characteristics of the cyst, as well as the surgeon’s expertise and patient-specific factors. After evaluating the disadvantages of the external open, rhinoplasty, and internal approaches in the literature, was made the decision to apply a new technique for our case. After completely removing the cyst from the entire nasal dorsum midline, the entire nasal dorsum defect was closed with a homogeneous tissue prepared using autologous septal cartilage and a mixture of tissue adhesives. A palisade-shaped dorsum was created to prevent nasal dorsum collapse. With this technique, was achieved a completely aesthetic and functional nasal shape. During the one-year follow-up period, no recurrence or aesthetic destruction was observed. This technique will have no skin defect, and reconstruction will be performed to minimize nasal deformity and cyst recurrence.

## 4. Conclusion

The purpose of introducing this technique in the literature is to increase recognition regarding the potential occurrence of nasal dermoid cysts on the midline nasal dorsum, even in later stages of life. It underscores the idea that these instances can be effectively addressed with minimized structural impact by means of meticulous surgical preparation.

## Data Availability

Data sharing does not apply to this article as no datasets were generated or analyzed during the current study.
